# Diagnostic and Therapeutic Potential of Extracellular Vesicles in B-Cell Malignancies

**DOI:** 10.3389/fonc.2020.580874

**Published:** 2020-09-29

**Authors:** Ernesto Gargiulo, Pablo Elías Morande, Anne Largeot, Etienne Moussay, Jérôme Paggetti

**Affiliations:** ^1^Tumor-Stroma Interactions, Department of Oncology, Luxembourg Institute of Health, Luxembourg, Luxembourg; ^2^Instituto de Medicina Experimental (IMEX)-CONICET-Academia Nacional de Medicina, Buenos Aires, Argentina

**Keywords:** extracellular vesicles, exosome, CLL, leukemia, lymphoma, myeloma, EV-based therapy

## Abstract

Extracellular vesicles (EV), comprising microvesicles and exosomes, are particles released by every cell of an organism, found in all biological fluids, and commonly involved in cell-to-cell communication through the transfer of cargo materials such as miRNA, proteins, and immune-related ligands (e.g., FasL and PD-L1). An important characteristic of EV is that their composition, abundance, and roles are tightly related to the parental cells. This translates into a higher release of characteristic pro-tumor EV by cancer cells that leads to harming signals toward healthy microenvironment cells. In line with this, the key role of tumor-derived EV in cancer progression was demonstrated in multiple studies and is considered a hot topic in the field of oncology. Given their characteristics, tumor-derived EV carry important information concerning the state of tumor cells. This can be used to follow the outset, development, and progression of the neoplasia and to evaluate the design of appropriate therapeutic strategies. In keeping with this, the present brief review will focus on B-cell malignancies and how EV can be used as potential biomarkers to follow disease progression and stage. Furthermore, we will explore several proposed strategies aimed at using biologically engineered EV for treatment (e.g., drug delivery mechanisms) as well as for impairing the biogenesis, release, and internalization of cancer-derived EV, with the final objective to disrupt tumor–microenvironment communication.

## Introduction

Extracellular vesicles (EV) are vesicular components released by every cell of an organism. This broad group consists of small EV or exosomes (sEV; 30–150 nm) characterized by an endocytic origin, microvesicles (MV; 100 nm–5 μm) shed from the plasma membrane, and apoptotic bodies (<5 μm) derived from membrane disintegration (1). The study of EV is a process in constant evolution, where EV characteristics, as well as nomenclature and isolation, are in continuous improvement (2–5).

Extracellular vesicles can be virtually found in every biofluid, which makes them relatively easy to recover and analyze (6). In the early years of EV-focused research, their synthesis and release were considered as a mechanism to remove harming material from the cell (7). Nowadays, this notion has been replaced by considering the mechanism as an active way to transfer material to targeted cells (1). Indeed the most common and accepted role of EV is intercellular communication through ligand–receptor interactions and the transfer of molecular cargoes.

The composition of EV is extremely heterogeneous. Depending on their nature, origin, physiological context, and parental cells, EV contain and transfer distinct elements to targeted cells, such as proteins, nucleic acids, lipids, metabolites, and organelles (8–10). The interest in understanding the role of EV in cancer is due to their unique ability to re-educate and attenuate the normal activity of healthy cells across the whole body. In line with this, B-cell malignancy-derived EV have been shown to interact with the surrounding microenvironment, leading to its profound remodeling. EV-based communication assists cancer development by stimulating tumor cell proliferation and migration (11), by modifying distant microenvironment to generate a pre-metastatic niche (12), and by strategically inhibiting tumor immune surveillance and anti-tumor response (13, 14).

## EV Diagnostic and Therapeutic Potential

**FIGURE 1:**
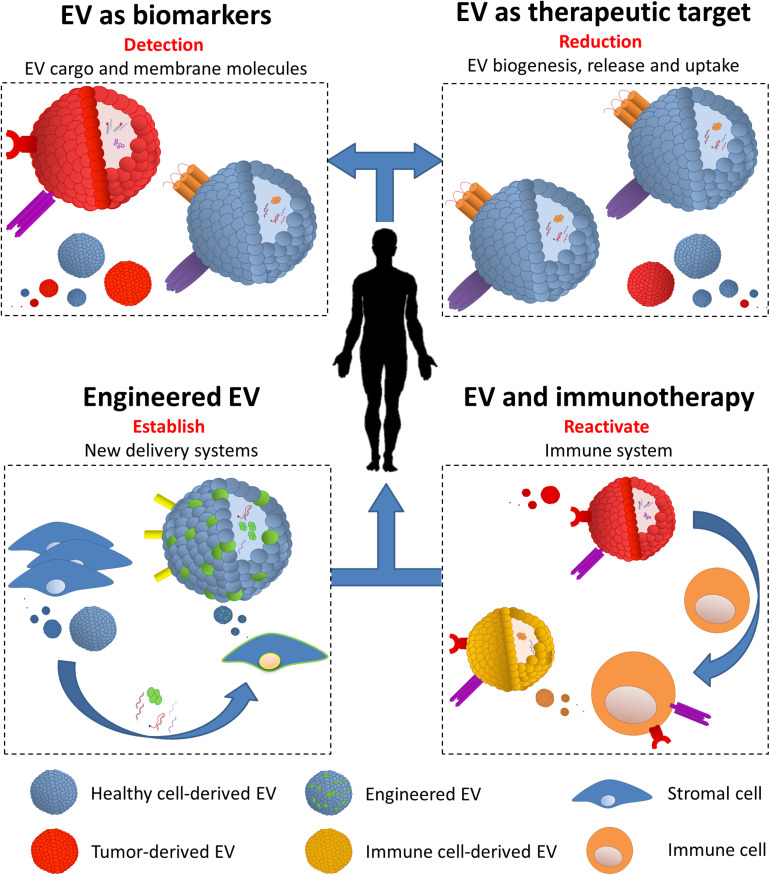
Extracellular vesicles (EV) diagnostic and therapeutic potential. (i) EV as biomarkers: multiple physiological characteristics allow EV to be easily collected from different biological sources (e.g., plasma and urine). Definition of content and carried molecules is essential to determine their origin, thus detecting tumor in different stages of the disease and in post-treatment follow-ups. (ii) EV as therapeutic targets: biogenesis, release, and uptake are all key steps which can be targeted in order to decrease the circulating amount and the function of tumor-derived EV. Beyond this, targeting active EV components (e.g., immune checkpoint) decreases their impact on various aspects on targeted cells. (iii) EV and immunotherapy: tumor cells have the ability to re-educate the immune system via the release of EV, thus able to affect the function of multiple immune cells. Despite this, tumor-derived EV can be strategically used to build a strong immune response by the endogenous immune cells (e.g., dendritic cells) and use their EV to diminish cancer cell functions. (iv) Engineered EV: together with nanoparticles, EV can be modified to carry specific molecules (e.g., shRNA and chemotherapy drugs) and directed to cancer and its microenvironment cells. Engineered EV can be produced by transformed cell lines (e.g., mesenchymal stem cells) and directed against tumor cells.

Due to a complex and parental-cell-dependent molecular cargo and to their presence in every biological fluid, EV represent an innovative tool for the design of diagnostic and therapeutic strategies in B-cell malignancies. In the sections below, we will explore the multiple potential uses of EV: (i) EV as biomarkers, (ii) EV as therapeutic targets, (iii) EV in immune evasion and use in immunotherapy, and (iv) biologically engineered EV (Figure 1 and Table 1).

**TABLE 1:** Overview of the diagnostic and the therapeutic potentials of extracellular vesicles in B-cell malignancies.

EV Role	Target	B-Cell malignancy	References
Biomarker	miR-21, miR-155, miR-146a, miR-148a, and Let-7g	CLL	(29–31)
	miR-24-3p, miR-127-3p, miR-21-5p, miR155-5p, and Let7a-5p	HL	(32)
	miR-18a and Let7b	MM	(33)
	CD19, CD20, CD5, CD37, CD52, and MHC	CLL	(36, 37)
	CD38, CD138, CD147, and CD44	MM	(39, 40)
Target	EV release (indomethacin)	DLBCL	(42)
	EV uptake (low molecular weight heparin)	CLL	(30)
	TGF-β1 (shRNA)	ALL	(44, 71)
Immune modulator	T lymphocytes (DC-derived EV) and DC (poly I:C)	ALL	(58)
	TGF-β1 (shRNA)	ALL	(44, 71)
Vaccination	DC (DLBCL-derived EV)	DLBCL	(78)
	T lymphocytes (DC-derived EV)	ALL	(64)
Carrier	c-Myc (siRNA)	Lymphoma	(70)
	TGF-β1 (shRNA)	Lymphoma	(44, 71)
	CD21 (Gp350)	CLL	(72)
	Apoptosis (Apo2L/TRAIL)	MM	(73)

*CLL, chronic lymphocytic leukemia; DLBCL, diffuse large B-cell lymphoma; MM, multiple myeloma; ALL, lymphocytic leukemia; HL, Hodgkin lymphoma.*

### EV as Biomarkers

Given that EV are continuously released by all the cells and that they can be recovered from every fluid in the body, they clearly represent a potential source of biomarkers (15, 16). Compared with most common biomarkers obtained from liquid biopsies, such as circulating tumor DNA (ctDNA), EV represent a more reliable source of information and can be easily incorporated in the diagnostic routine. Specifically, ctDNA represents only a minimal fraction of plasma-cell-free DNA (17, 18); thus, despite its high specificity, the starting amount is already challenging (17, 19). Furthermore, ctDNA is often affected by high fragmentation and low stability (20, 21). EV are very stable and, depending on the time of use, can be stored at different temperatures for extended periods of time (several weeks at 4°C, several months at −80°C, and several years in liquid nitrogen) (22). Cancer cells generally produce high amounts of EV that mirror the characteristics—as a sort of fingerprint—of the parental cancer cells (23–25). Indeed cargo composition (e.g., microRNA and proteins) and transmembrane molecules (tumor antigens or immune cell markers) allows the identification of the parental cell and its physiological state (24, 26). Analysis of EV isolated from patients was confirmed to be an important tool in the detection of cancer development at an early stage and to stratify patients based on intrinsic tumor characteristics and for following patients in remission (16, 27, 28).

In line with this, we have shown that the plasma of a patient affected by chronic lymphocytic leukemia (CLL) is highly enriched in multiple key microRNAs (e.g., miR-21, miR-155, and miR-146a) which can potentially affect multiple pathways involved in the pathogenesis and the progression of the disease. MicroRNA expression profile can also be used to stratify CLL patients in view of a more personalized treatment and follow-up (29). Importantly, we and others found these same microRNAs and some additional ones (miR-148a and Let-7g) also in CLL-derived EV, demonstrating how these vesicles can indirectly provide information on cellular state (30, 31). Van Eijndhoven and colleagues detected a higher level of EV carrying miR-24-3p, miR-127-3p, miR-21-5p, miR155-5p, and Let7a-5p in the plasma of Hodgkin lymphoma patients compared with controls. Upon treatment, patients showing remission had a stable reduction of these miRNAs compared with relapse patients who displayed their progressive accumulation (32). Interestingly, a further study performed on a large multiple myeloma (MM) cohort described that the progression-free survival and the overall survival of patients could be successfully followed by monitoring the plasma levels of miR-18a and Let7b, which were found to be highly enriched in MM-derived EV (33).

Beyond miRNA content, RNA packed in plasma EV has the potential to be used to define tumor mutation landscape, such as BRAF V600E for melanoma, with the aim to detect early-stage disease or define the best clinical approach for certain patients (34). This strategy may be transposed to mutated RNA contained in circulating EV from B-cell malignancies, with the attempt to perform a personalized patient follow-up and therapy.

EV reflect parental cell also through the presence of cellular markers on the EV membrane and in their protein cargo (35). Different studies show that CLL-derived EV from a patient’s plasma present typical markers of leukemic cells (e.g., CD19, CD20, CD5, and CD37) and major histocompatibility complex (e.g., HLA-A, HLA-B, and HLA-C) (36, 37). Despite this correlation, it is essential to highlight that not all markers or their possible combinations are always present on EV released by tumor cells. Indeed CLL cells can release a subset of EV carrying CD52 but only few CD19 molecules; this particular marker combination was shown to be characteristic of CLL patients with advanced disease (38). Another study by Harshman and colleagues showed the presence of typical MM cell (CD38, CD138, and CD147) and treatment resistance (CD44)-associated markers on MM-derived EV isolated from a patient’s serum (39, 40).

In sum, EV represent a faithful snapshot of the parental cell that synthesized them. The follow-up of proteins and RNAs carried by circulating EV in B-cell malignancy patients constitutes a promising source of predictive biomarkers. The low invasiveness of their isolation could allow regular sampling, and by monitoring EV content, the evolution of tumors within patients can be followed, either before or after anti-cancer treatment, allowing clinicians to select the best course of action for each specific case.

### EV as Therapeutic Targets

Biogenesis, release, and internalization of EV are tightly regulated processes that involve multiple putatively targetable protein complexes. Impairing the pro-tumoral effect of EV can be achieved by acting through two main axes. On one hand, the autocrine signals, where EV bind to and modify neoplastic cells themselves, could be disrupted (41). On the other hand, the role of EV in the cross-talk between cancer cells and the supportive microenvironment could be targeted. This can have a major impact on decreasing tumor survival, proliferation, and migration (42).

In B-cell malignancies, Koch and colleagues demonstrated that treating several diffuse large B-cell lymphoma (DLBCL) cell lines with indomethacin, a non-steroidal anti-inflammatory drug, suppressed EV release and strongly decreased tumor progression. They further demonstrated that a decreased EV release guarantees a more potent effect of cytostatic drugs, such as anthracyclines and anthracenediones, both *in vitro* and *in vivo* (42). Another strategy implies the reduction of EV uptake by blocking key factors, e.g., heparan sulfate proteoglycans (HSPGs), on target cells (41). HSPGs have been suggested to act as a receptor for EV internalization. In line with this, we demonstrated that pre-treatment of EV with low molecular weight heparin, a HS analog, strongly decreases the uptake of CLL-derived EV by target cells (30).

Several studies have demonstrated how EV have a strong impact on the immune system, allowing a more efficient tumor immune evasion (13). One of the characteristics associated with this phenomenon is the presence of immune checkpoint (ICP) ligands on tumor-derived EV. Chen et al. (43) recently showed an increase in PD-L1 molecule on melanoma derived-EV upon interferon-γ stimulation. Considering that PD-L1 and, potentially, other ICP molecules are present on EV derived from B-cell malignancies characterized by a strong immune-suppressed microenvironment (e.g., CLL), therapies such as recombinant blocking antibodies can represent an effective solution in targeting immune-suppressive EV. The same strategy can also be extended to other EV molecules; for instance, targeting leukemia-derived EV bearing transforming growth factor-β1 (TGF-β1) has been shown to improve anti-leukemia immunity (44).

Molecules carried by tumor-derived EV are not the only source of harming. EV released by tumor microenvironment B-cells carry CD39 and CD73, two surface molecules able to hydrolyze ATP released by dying cancer cells to adenosine and effectively hijack CD8 T cell immune activity by binding the A2A adenosine receptors (45). In line with this, it is possible to speculate that B-cell malignancy-derived EV may have a similar effect, if not greater, given their higher concentration in the tumor milieu. The study of Zhang et al. (45) shows that a decrease of B-cell-derived EV bearing CD73 and CD39 can be achieved by deregulating the docking protein RAB27A. This has been performed using an inactivated Epstein–Barr virus-mediated siRNA, but it is also possible to generate EV (e.g., derived from modified stroma cell lines) carrying RAB27A siRNA and specifically deliver it to tumor cells.

Targeting B-cell malignancy EV represents an interesting strategy in the treatment of cancer. These approaches imply obvious risks and limitations, such as possible drug resistance and off-targets, which can ultimately lead to decreased efficacy of the therapy.

### Role of EV in Immune Evasion and Use in Immunotherapy

The immune system is one of the main players involved in cancer cell recognition and elimination (46–48). However, cancers deploy numerous strategies to repress the immune system during disease development and, thus, effectively evade immune surveillance. The concept behind immunotherapy is to re-activate the patient’s immune system in order to recognize and remove cancer cells. One example of the many mechanisms involved in immunoevasion is the use of EV to fully re-educate the immune microenvironment, causing a domino effect and ultimately leading to overall immune repression and cancer development (13).

Being released by neoplastic cells, EV represent an important source of selective antigens that educate naïve immune cells (49, 50). As for solid tumors (51–53), leukemia-derived EV are loaded with antigens and several immunogenic molecules, such as TGF-β and IL-6, capable to impair dendritic cells (DCs) to build a specialized immune response against neoplastic cells (54–57). EV structure and characteristics allow them to specifically drive DC-dependent immunization; in fact, stimulation of DCs with only leukemic cell lysate results in failure to build an appropriate immune response (55, 56).

Immunotherapy can also be used in parallel with chemotherapy. Guo and colleagues combined leukemia-specific DC-derived EV with cyclophosphamide and polyinosinic-polycytidylic acid sodium salt (poly I:C) (58). This combination is based on the ability of DC-derived EV to stimulate T lymphocyte proliferation and enhance their cytotoxic activity against leukemia, while poly I:C acts on DC maturation. This strategy led to leukemia cell suppression *in vitro* and increased survival of tumor-bearing mice (58).

Targeting tumor-derived EV surface molecules is also a valuable strategy to strongly reduce their activity on immune cells. As we have previously mentioned, TGF-β1 is enriched in EV released by a wide range of tumors and, acting on various immune cells such as DCs, is one of the main causes of immune escape (57). EV released by acute lymphocytic leukemia (ALL) L1210 cell line carry high levels of TGF-β1. By using small hairpin RNA (shRNA), Huang and colleagues knocked down TGF-β1 in the ALL cell line, thus removing it from their EV and reconstituting DC maturation and activity *in vivo*. Additionally, the same group demonstrated that pulsed DCs were able to increase T cell proliferation and enhance cytotoxic activity against ALL cells (44).

Dendritic cells are not the only immune cells that can be used to re-establish a proper cytotoxic activity against B-cell malignancies. Another valid target is natural killer (NK) cells, whose activity is repressed by molecules, such as NKG2D ligands, exposed on hematological malignancy (HM)-derived EV (59, 60). One particular study presented a double role of the NKp30 ligand BAG6 in CLL. First, high levels of soluble BAG6 can be detected in the plasma of CLL patients, being one of the causes for the defect in NK cytotoxicity observed in these patients (61). At the same time, BAG6 is released *via* EV by healthy cells in response to cellular stress and has a protective activity by enhancing NK cytotoxicity (60). Based on this, it is possible to hypothesize that treatment with BAG6^+^ EV derived from stressed cells may be a reliable opportunity to maintain (and possibly to re-establish) NK activity during cancer development.

Despite their evident activation effects on the immune system, the use of EV-based vaccination is still under discussion, although several studies already suggest how EV can be used to build an effective immune response before tumor cells arise. In line with this, DC cells pulsed with DLBCL-derived EV have been shown to stimulate T lymphocyte expansion and, by consequence, increase anti-lymphoma immunity in mice (62). These results are in accordance with other studies where leukemia-derived EV can be used to build anti-leukemia immunity, with evident results both *in vitro* and *in vivo* (54, 63). Finally, Qazi and colleagues showed that EV antigens alone are enough to induce memory T lymphocytes through B-cell activation (64).

Altogether these findings position EV as potent immune modulators that can become a major tool for immunotherapy and vaccine design.

### Engineered EV

The ability of EV to bind to specific receptors on both tumor and microenvironment cells through surface molecules makes them an interesting tool to transfer exogenous cytotoxic and inhibitory molecules for therapeutic purposes (65). Furthermore, EV represent a useful method to deliver anti-tumor drugs due to their ability to retain stable concentrations of the components loaded in them as well as a natural accumulation in vascular sites, such as inflammation and wounds, tumor, and infection areas (66–69).

Lunavat and colleagues successfully engineered EV-like nanoparticles (NV) that contain siRNA and showed that silencing c-Myc by this approach efficiently activates poly (ADP-ribose) polymerase-dependent apoptotic pathways in treated λ820 lymphoma cells (70). More recently, shRNA strategy has been used to silence TGF-β1 in lymphoma cells, forcing them to release TGF-β1-depleted EV. By removing this strong antitumor–immune surveillance inhibitor, the authors achieved an increase in the response of the immune system against leukemic cells (71). In line with this, engineered EV were also used to transfer specific antigens, with the purpose of re-activating or enhancing the immune system. Stromal cells transfected with the Epstein–Barr virus (EBV) protein gp350 release gp350^+^ EV that specifically interacts with CD21 on B-cells. Gp350^+^ EV were further engineered to carry CD154. By treating CLL patients’ cells, the authors achieved the internalization of EV in leukemic cells and a strong immunogenic effect, leading to a dual activation of tumor-associated and EBV-specific T cells (72).

Further strategies based on EV-like nanostructures have been explored in B-cell malignancies. Artificial lipid vesicles (ALV) decorated with bioactive Apo2 ligand/TNF-related apoptosis-inducing ligand were tested on lymphoma and MM cell lines. These novel ALV showed a consistent pro-apoptotic effect in multiple HM cell lines while sparing normal cells (such as CD4^+^ and CD8^+^ T cells) *in vitro* and with no sign of toxicity *in vivo* (73).

Engineered EV can be broadly produced *in vitro* using a wide range of cell lines. Mesenchymal stem cells (MSCs) have been shown to be a valuable tool for the production of EV with high tropism toward tumor cells. In line with this, MSCs can be modified to release engineered EV with various downstream applications and means (74).

As mentioned before, ICPs on EV surface are important targets for immunotherapy. As alternative to monoclonal antibodies, EV released by modified MSCs can be used as antagonists for delivering immune checkpoint blockade proteins (e.g., ICP receptors or monoclonal antibodies) (75). A further well-known immune escape strategy applied by tumors is the overexpression of CD47, also carried by tumor-derived EV, which interacts with the signal regulatory protein α (SIRPα) on phagocytic cells, limiting their activity (76). The production of EV bearing SIRPα molecules has been shown to successfully hamper the tumor “don’t-eat-me” signal in favor to macrophage phagocytosis (77).

Recently, a new class of engineered EV has been tested, with the aim to enhance T cell activity against tumor cells. Synthetic multivalent antibodies retargeted (SMART) EV bear monoclonal antibodies against CD3 and cancer cell-associated epidermal growth factor receptor (EGFR). These characteristics allow them to act as a bridge directing T cytotoxic cells toward tumor cells, thus inducing crosstalk and enhancing antitumor response (78). SMART EV may have a potential application on MM due to its high level of EGFR, which guarantee proliferation and resistance to conventional therapies (79, 80). Furthermore, the same strategy could be applied for other B-cell malignancies, such as CLL and lymphoma, by targeting highly enriched surface molecules.

EV are also able to epigenetically reprogram target cells and to completely change their phenotype in a short time (81, 82). A potential strategy in the re-activation of the immune system can be to inhibit the molecules responsible for this deep change as well as creating engineered EV carrying specific cargo components (e.g., siRNA and shRNA) that are able to lift the immune cell exhaustion by rewiring the epigenetic landscape.

An interesting application for engineered EV is to use them as an instrument for decoy of cytokines (e.g., pro-inflammatory cytokines). This strategy actively restrains tumor effect on microenvironment cells (e.g., reducing inflammation). An example comes from a recent preprint where stroma-derived EV were engineered to bear tumor necrosis factor receptor 1 and interleukin 6-signal transducer. The authors reported striking effects concerning inflammation reduction and survival of experimental mouse models (83). A possible application of this strategy in B-cell malignancies would be to decoy important cytokines related to immune suppression, such as IL-10 and TGF-β1 (44, 84).

Various studies have successfully used EV as a drug delivery system. The rationale to generate EV containing specific drugs is based on cell ability to encapsulate exogenous material and release either actively (e.g., microvesicles budding) or by cellular death consequence (apoptotic bodies). Based on this, EV released by drug-treated tumor cells have been used to deliver specific chemotherapeutic agents to untreated tumor cells (85). EV-based drug delivery has been tested on various cancer models, including multiple drug resistance (MDR). In each case, EV shielded the therapeutic agents and delivered them to tumor cells, demonstrating a higher cytotoxic effect compared with its administration alone (85, 86). Furthermore, Osterman et al. (87) have shown how EV can be used to encapsulate toxic drugs, such as curcumin, and be specifically directed to cancer cells, highly reducing any side effect. Despite the fact that no EV-based drug delivery has been established for B-cell malignancies, strategies to encapsulate chemotherapeutic agents should be explored. One of the many possibilities could be to encapsulate the ceramide supplement C6 in EV to target MM cells. Indeed Chang and colleagues showed *in vitro* how C6 ceramide treatment leads to increased apoptosis and block the proliferation of MM cells by upregulating miRNA clusters such as miR-202 and miR-16 (88).

Engineered EV, as well as artificial NV, are powerful and plastic tools to be deployed against B-cell malignancies. As mentioned above, it is evident that one of the multiple advantages lies in the possibility to combine this strategy with other well-established approaches, such as those aiming at reactivating the host immune system. Furthermore, using engineered EV with a tropism toward specific cells may improve the targeting of tumor EV. Loading shRNA into these delivery systems has the potential to reduce tumor EV production, release, and uptake. Finally, it is important to consider the high affinity of engineered EV for tumor cells as a strategic tool to deliver chemotherapy drugs, thus reducing any off-target side effects.

## Ongoing Clinical Trial Using EV

Several clinical trials involving EV are currently ongoing. In cancer, the vast majority are applied to solid tumors. The aims are various: to establish novel sources and standardized methods to isolate EV from patients (NCT03821909), to deepen the characterization of cancer EV as predictive biomarkers (NCT03830619), to develop EV-based vaccination (NCT01159288), and finally to further improve EV-based treatment (NCT03608631).

In reference to B-cell malignancies, the ExoReBly project (NCT03985696) has the overall aim to characterize DLBCL-derived EV from patients’ samples. The rationale of this clinical trial is based on the fact that half of the patients subjected to immunotherapy—typically with the aim to stimulate immune activity against CD20—fails to show any benefit. The major hypothesis is that the presence of a high amount of CD20 and PD-L1, present on DLBCL-derived EV, acts as a decoy target for rituximab antibody and as a strong immunosuppressive signal, respectively, leading to therapeutic resistance. Apart from the characterization of DLBCL-derived EV, the project aims to use these EV as a marker of response to therapy and disease outcome. Interestingly, we made a similar observation with CLL-derived EV presenting high levels of CD20 on their surface and being able to act as a decoy for rituximab, potentially highlighting a conserved mechanism among B-cell malignancies (30).

With over 70 clinical trials, EV are gaining more attention in view of practical and clinical applications. A small percentage of these clinical trials take in consideration HM, and only one is being developed in B-cell malignancies. Nevertheless, every year, new information concerning EV impact on cancer progression is generated, which strongly suggests that more applications for HM will arise.

## Conclusion

The path to decipher the complex characteristics of EV composition in B-cell malignancies, as well as the biological role that they accomplish in the behavior of tumors, has shown important advances in the last decade. The increasing amount of novel data recently generated reflects a continuous interest for studying EV within the community, due in part to their potential as a tool to improve cancer diagnosis and therapy. It is now well established that EV can mirror the tumor cells they originate from and can carry key targetable molecules strongly related to cancer biology, like immune checkpoints among many others. Consequently, several additional important discoveries herein summarized pinpoint a central role attributed to EV in B-cell driven diseases diagnosis and follow-up and for putative novel treatment strategies.

However, these reports remain in the area of translational research but are not yet successfully translated to clinical applications for patients with B-cell malignancies. While the treatment of solid cancers using an EV-derived rationale is already ongoing in advanced clinical trial phases, this is neither the case for the diagnosis and the follow-up nor for any treatment in B-cell-originated neoplastic diseases. The fact that EV-based approaches work properly in other chronic neoplastic and non-neoplastic diseases represents a motivation to fully enhance initiatives that use this rationale in HM in the near future. Thus, the upcoming decade will hopefully shed light into the clinical applicability of EV as a powerful tool for patients with B-cell neoplastic disorders.

## Author Contributions

EG wrote the manuscript and created the figure. PM and AL helped in writing the manuscript. EM and JP finalized the manuscript and supervised the team. All authors contributed to the article and approved the submitted version.

## Conflict of Interest

The authors declare that the research was conducted in the absence of any commercial or financial relationships that could be construed as a potential conflict of interest.

## Abbreviations

ALL: acute lymphocytic leukemia
ALV: artificial lipid vesicles
BTK: Bruton tyrosine kinase
CLL: chronic lymphocytic leukemia
ctDNA: circulating tumor DNA
CTX: cyclophosphamide
DC: dendritic cell(s)
DLBCL: diffuse large B-cell lymphoma
EBV: Epstein–Barr virus
EGFR: epidermal growth factor receptor
EV: extracellular vesicle(s)
HL: Hodgkin lymphoma
HM: hematological malignancy
HS: heparan sulfate
ICP: immune checkpoint
IFN- γ: interferon- γ
IL6ST: interleukin 6-signal transducer
MDR: multiple drug resistance
MSC: mesenchymal stem cell(s)
MM: multiple myeloma
MV: microvesicle(s)
NK: natural killer(s)
NV: nanoparticle(s)
PG: proteoglycans
sEV: small extracellular vesicle(s)
shRNA: small hairpin RNA
SIRP α: signal regulatory protein α
SMART: synthetic multivalent antibodies retargeted
TGF- β (1): transforming growth factor- β (1)
TNFR1: tumor necrosis factor receptor 1.
